# The microRNA miR-10b as a potentially promising biomarker to predict the prognosis of cancer patients: a meta-analysis

**DOI:** 10.18632/oncotarget.21428

**Published:** 2017-09-30

**Authors:** Yi Zhang, Rong-Bo Liao, Li-Lin Hu, Bi-Xia Tong, Teng-Fei Hao, Hua-Jun Wu

**Affiliations:** ^1^ Department of General Surgery, The First People’s Hospital of Neijiang, Neijiang 641000, Sichuan Province, P.R. China; ^2^ Department of General Surgery, The Second Affiliated Hospital of Nanchang University, Nanchang 330000, Jiangxi Province, P.R. China; ^3^ Department of Medicine, Nanchang University, Nanchang 330000, Jiangxi Province, P.R. China; ^4^ Department of Nursing, Shangrao People’s Hospital, Shangrao 334000, Jiangxi Province, P.R. China

**Keywords:** miR-10b, carcinoma, metastasis, meta-analysis

## Abstract

**Background:**

Reported studies on carcinoma have evaluated the significance of the microRNA miR-10b in the development and progression of many cancers. Increased expression of miR-10b is associated with the susceptibility to lymph node metastasis and distant metastasis in various tumors.

**Results:**

The results of the meta-analysis revealed that lymph node metastasis occurred more frequently in the patients group with high expression level of miR-10b than in the patients group with low expression level of miR-10b (OR=4.65, 95% CI: 3.40–6.37, *P* <0.00001, fixed-effects model). Additionally, a similar result was observed in the association between miR-10b expression and distant metastasis (OR=2.70, 95% CI: 1.79–4.08, *P* <0.00001, fixed-effects model).

**Materials and Methods:**

In this study, a meta-analysis, including the majority of the relevant articles, was conducted to investigate the association of the miR-10b expression level with metastasis in cancer patients. Systematic literature retrieval was performed by searching in a number of electronic databases. The meta-analysis was conducted using the RevMan 5.2 software and Stata SE12.0 software. A total of 962 patients with carcinoma from 9 studies were included in analysis.

**Conclusions:**

This meta-analysis demonstrated that the overexpression of miR-10b was significantly correlated with metastasis status, and indicated the potential clinical use of miR-10b as a molecular biomarker, particularly in assessing prognosis for patients with cancers.

## INTRODUCTION

According to a recent report, carcinoma is a major cause of mortality, accounting for the death of 8.2 million people and 14.1 million newly diagnosed cancer cases worldwide in the year 2102 [1]. The mechanism of tumor formation varies in different cancers, but carcinoma with metastasis is an important prognostic factor, and the presence or absence of metastasis determines treatment strategies [2, 3].

A majority of malignant tumor cases can ultimately develop metastases, which include lymph node metastases (LNM) and distant metastases (DM). Most cancers are diagnosed at an advanced stage, and the rate of lymph node metastasis or distant metastasis remains high. Until now, the precise metastasis mechanism of cancer cells is still unclear. In recent years, microRNAs (miRNAs), which act as gene expression regulators by regulating messenger RNA translation and degradation, have been found to have an effect on the occurrence and development of cancer. Accordingly, they have become a hot research topic, and their role in cancer is being recognized by more and more scientists. A better understanding of the molecular mechanism underlying carcinoma development, invasion and metastasis is of great clinical value to find reliable diagnostic markers and develop novel therapeutic strategies.

MicroRNA-10b (miR-10b) has been reported to be dysregulated in certain types of cancer and to play an important role in invasion and metastasis [4–6]. Previous studies show that miR-10b is up-regulated in breast cancer with distant metastasis, liver, pancreatic and esophageal cancers, and in glioma tissues [7–9]. Its expression was higher in cancerous tissues compared with paired noncancerous tissues or adjacent tissues. Also, some studies have demonstrated that the expression level of miR-10b was up-regulated in cancerous tissues compared to non-neoplastic tissue or adjacent tissue, and was associated with some clinical features, including LNM and DM. Moreover, there were associations between miR-10b expression and certain clinical characteristics of cancer patients. However, even though miR-10b may act as a common molecular marker for both LNM and DM, currently, we still know nothing about its role in predicting metastases of various cancers. Therefore, in the present study, the relevant literature was retrieved and evaluated according to the inclusion and exclusion criteria. The studies that met the criteria were included in this meta-analysis. The correlation between the expression level of miR-10b and metastasis status was analyzed and to further determine whether miR-10b could be a promising noninvasive biomarker to predict the prognosis of cancer patients.

## RESULTS

### Studies’ characteristics

**Figure 1:**
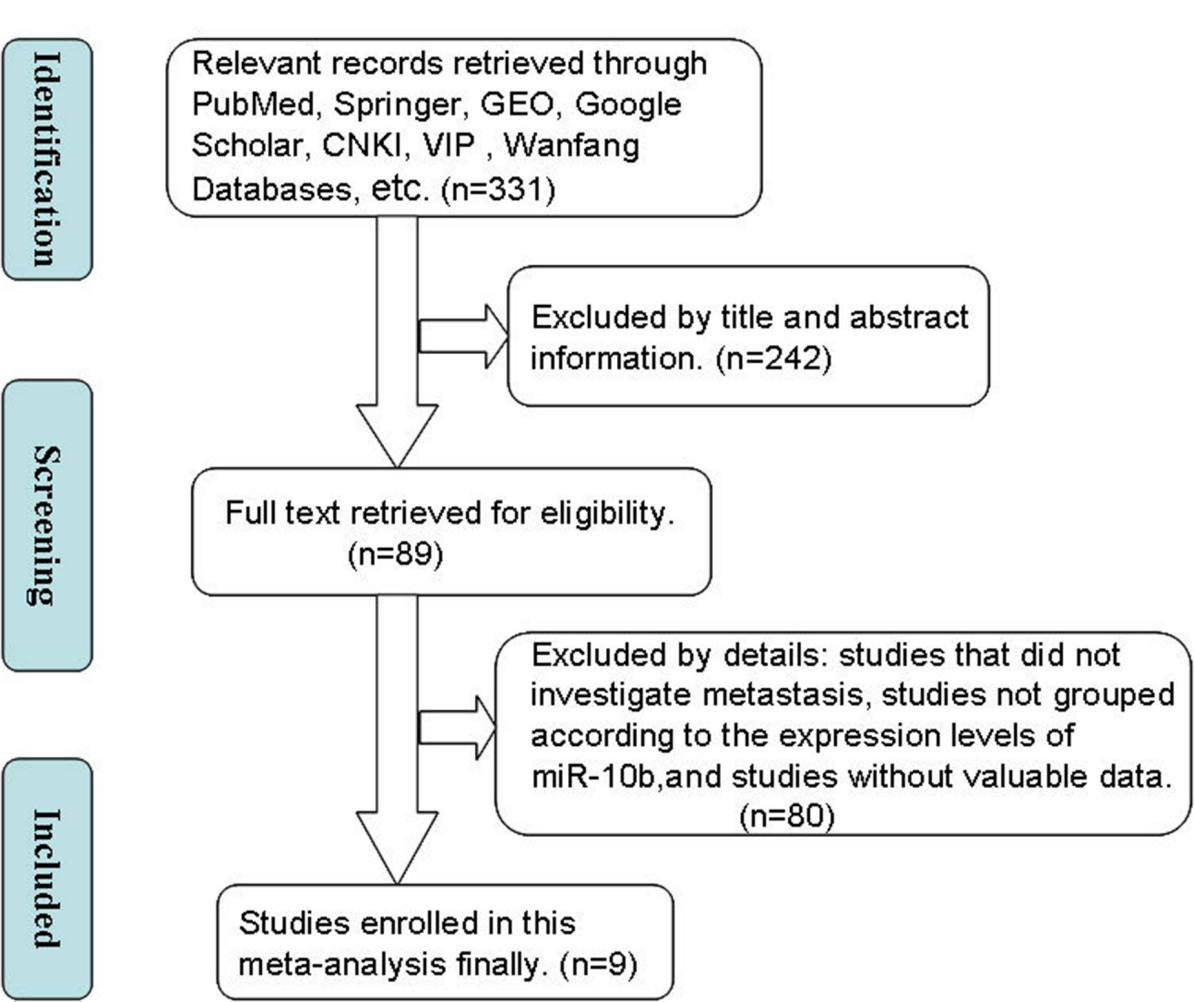
A flowchart presenting the steps of literature retrieval and selection CKNI, China National Knowledge Infrastructure; VIP, Chongqing VIP Information Network; GEO, Gene expression Omnibus**.**

The literature retrieval process is depicted in detail in Figure 1. A total of 9 studies [10–18] were ultimately found to be eligible according to the selection criteria and included in this meta-analysis. A total of 962 patients were included in the present meta-analysis, and the mean sample size of patients per study was 106.9 (range:34–436). Among the 9 studies, 8 were from the People’s Republic of China and one was from Japan. Five different cancer types were covered in this meta-analysis, including one breast cancer, five non-small cell lung cancer, one gastric cancer, one colorectal cancer and one hepatocellular carcinoma. All cancerous specimens were well preserved before RNA extraction. The diagnoses of LNM and DM were all based on pathological examination.

There were eight studies that reported on the association between the expression of miR-10b and LNM occurrence [10, 11, 13–18], and six articles focused on the association of miR-10b expression and distant metastases [10, 12–15, 17]. The detection methods, namely reverse transcription polymerase chain reaction was used to determine the expression levels of miR-10b in cancerous tissues. The criteria of high miR-10b expression in all included studies are shown in Table 1. All studies were divided into two groups (high miR-10b expression group and low miR-10b expression group).

**Table 1:** The basic information and data of all included studies in the meta-analysis

Author(year)	Country	Race	Cancer type	Total number	miR-10b expression				Detection method	Criterion of high expression
					High LNM/DM	High	Low LNM/DM	Low		
Nishida et al (2012) [13]	JAPAN	Asian	CRC	88	21/10	44	15/7	44	qRT PCR	above median
Luan et al (2014) [18]	CHINA	Asian	NSCLC	50	15/-	25	6/-	25	qRT PCR	RQ>1.18
Wang et al (2013) [14]	CHINA	Asian	GC	436	151/40	183	119/21	253	qRT PCR	Staining index score≥4
Zhang et al (2014) [16]	CHINA	Asian	NSCLC	73	21/-	37	5/-	36	qRT PCR	HOTAIR/GAPDH ≥1.0
Li et al (2012) [12]	CHINA	Asian	HCC	34	-/13	17	-/7	17	qRT PCR	above median
Li YW et al (2016) [11]	CHINA	Asian	NSCLC	58	23/-	25	21/-	33	qRT PCR	2^-∆Ct^≥0.003
Huang et al (2015) [10]	CHINA	Asian	NSCLC	114	45/7	55	34/6	59	qRT PCR	∆∆Ct>range/2
Yang et al (2015) [15]	CHINA	Asian	NSCLC	74	30/6	64	1/0	10	qRT PCR	RQ≥1.15
Cheng et al (2012) [17]	CHINA	Asian	BC	35	17/14	18	9/6	17	qRT PCR	Fold control >23.73

The dashes represent no data.

BC, breast cancer; CRC, colorectal cancer; HCC, hepatocellular carcinoma; NSCLC, non-small cell lung cancer; GAPDH, glyceraldehyde 3-phosphate dehydrogenase; GC, gastric cancer; DM, distant metastases; LNM, lymph node metastases; RT-PCR, reverse transcription polymerase chain reaction; RQ=2^-∆∆CT^; Staining index score:staining intensity score and the proportion of positive tumor cells.

### Association between miR-10b expression level and LNM

**Figure 2:**
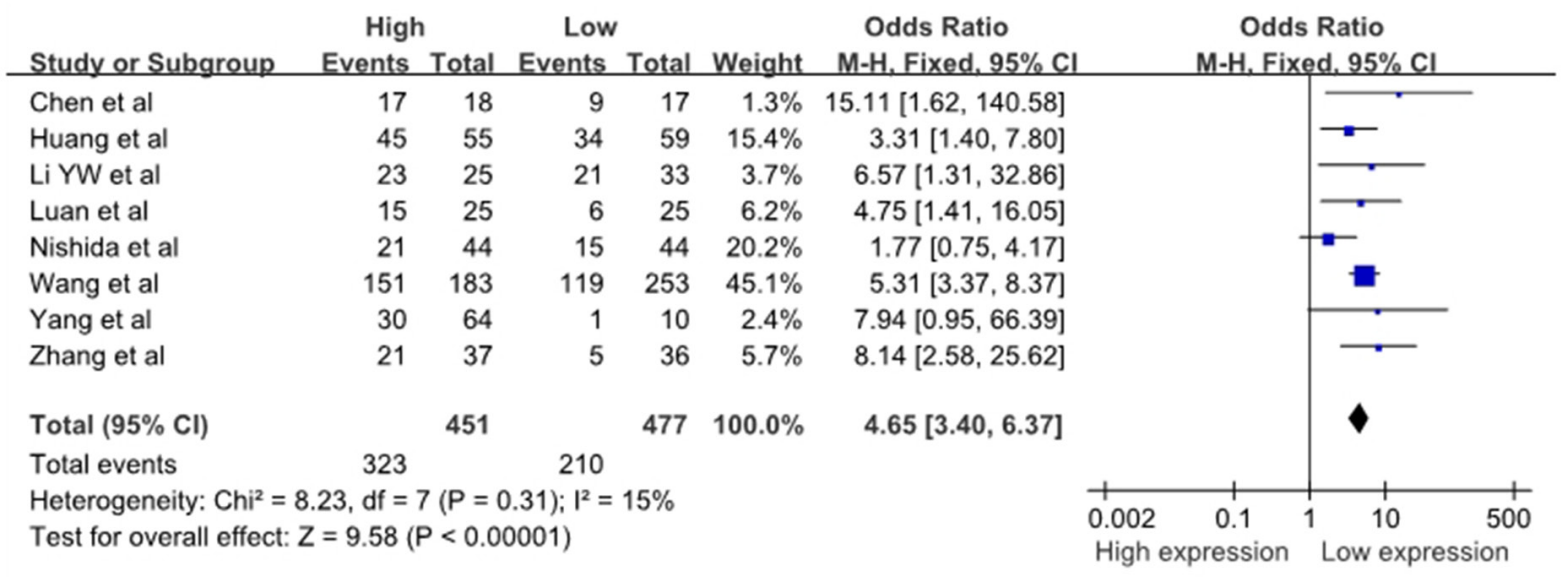
A forest plot for the association between the miR-10b expression levels with LNM LNM, lymph node metastases; M–H, Mantel–haenszel test; df, degrees of freedom.

Eight studies reported the number of patients with LNM based on different miR-10b expression levels, which comprised 928 cases. The fixed-effects model was adopted because there was limited heterogeneity across studies (I^2^=15%, P_Q_=0.31). The odds ratio (OR), expressed as the high miR-10b expression group versus low miR-10b expression group, was 4.65 (95%CI: 3.40–6.37, P<0.00001) is shown in Figure 2. When the LNM incidence in the cancer patients was compared between the two groups, the results of the meta-analysis revealed that the difference in the LNM incidence between the high and low expression groups was statistically significant. This result demonstrated that cancer patients with high miR-10b expression in cancerous tissues were more prone to develop LNM.

### Association between miR-10b expression level and DM

**Figure 3:**
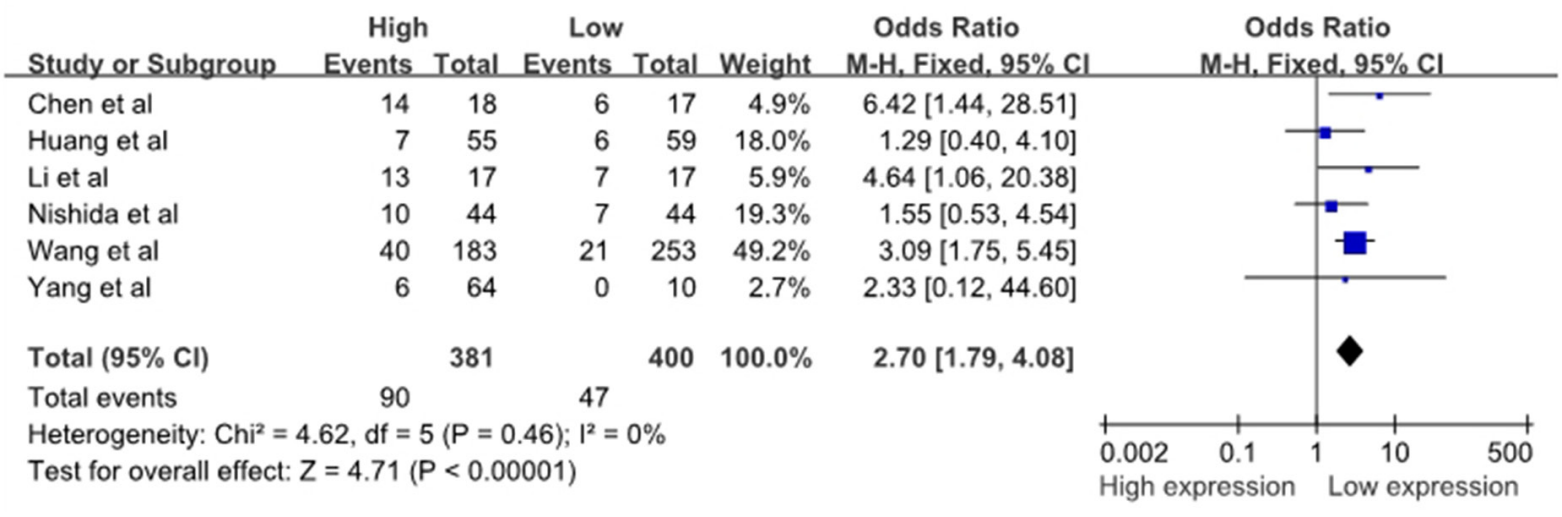
A forest plot for the association between the miR-10b expression levels with DM DM, distant metastases; M–H, Mantel–haenszel test; df, degrees of freedom.

Six studies reported the number of patients with DM based on different miR-10b expression levels, which comprised 781 patients. There was no significant heterogeneity among the studies (I^2^=0%, P_Q_=0.46); thus, the fixed-effects model was adopted. The analysis showed a pooled OR =2.70 (95% CI: 1.79–4.08, P<0.00001), as shown in Figure 3. Compared with the low miR-10b expression group, the DM rate was significantly increased in the high miR-10b expression group. The result revealed that the high miR-10b expression level in the patients’ tumor tissues may be an increased risk of developing DM. It must be mentioned that, due to the relatively small heterogeneity across studies on distant metastasis and the limited number of included studies, the sensitivity analysis and assessment of publication bias were not performed.

### Publication bias

**Figure 4:**
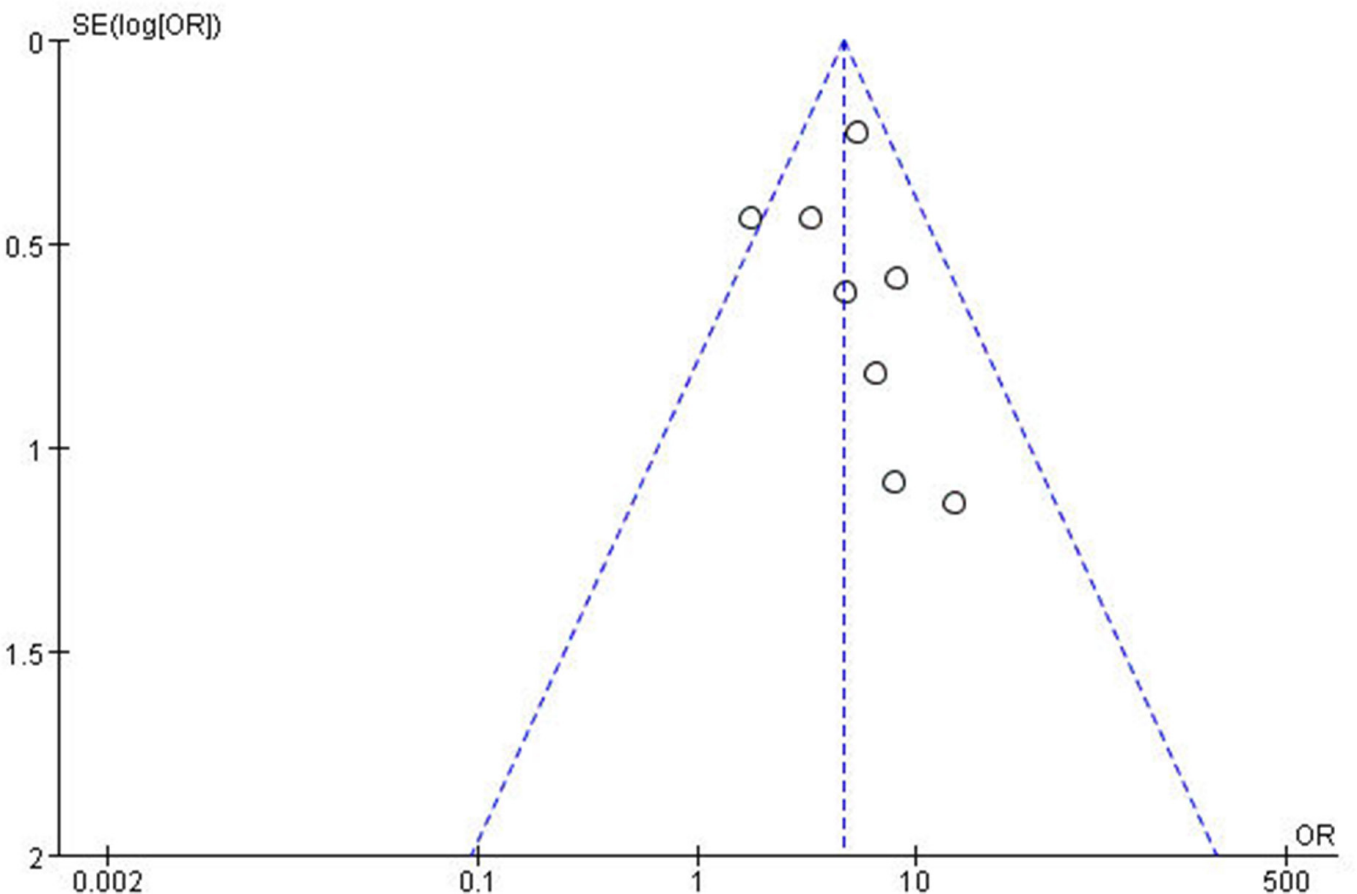
A funnel plot analysis of potential publication bias SE, standard error; OR, odds ratio.

**Figure 5:**
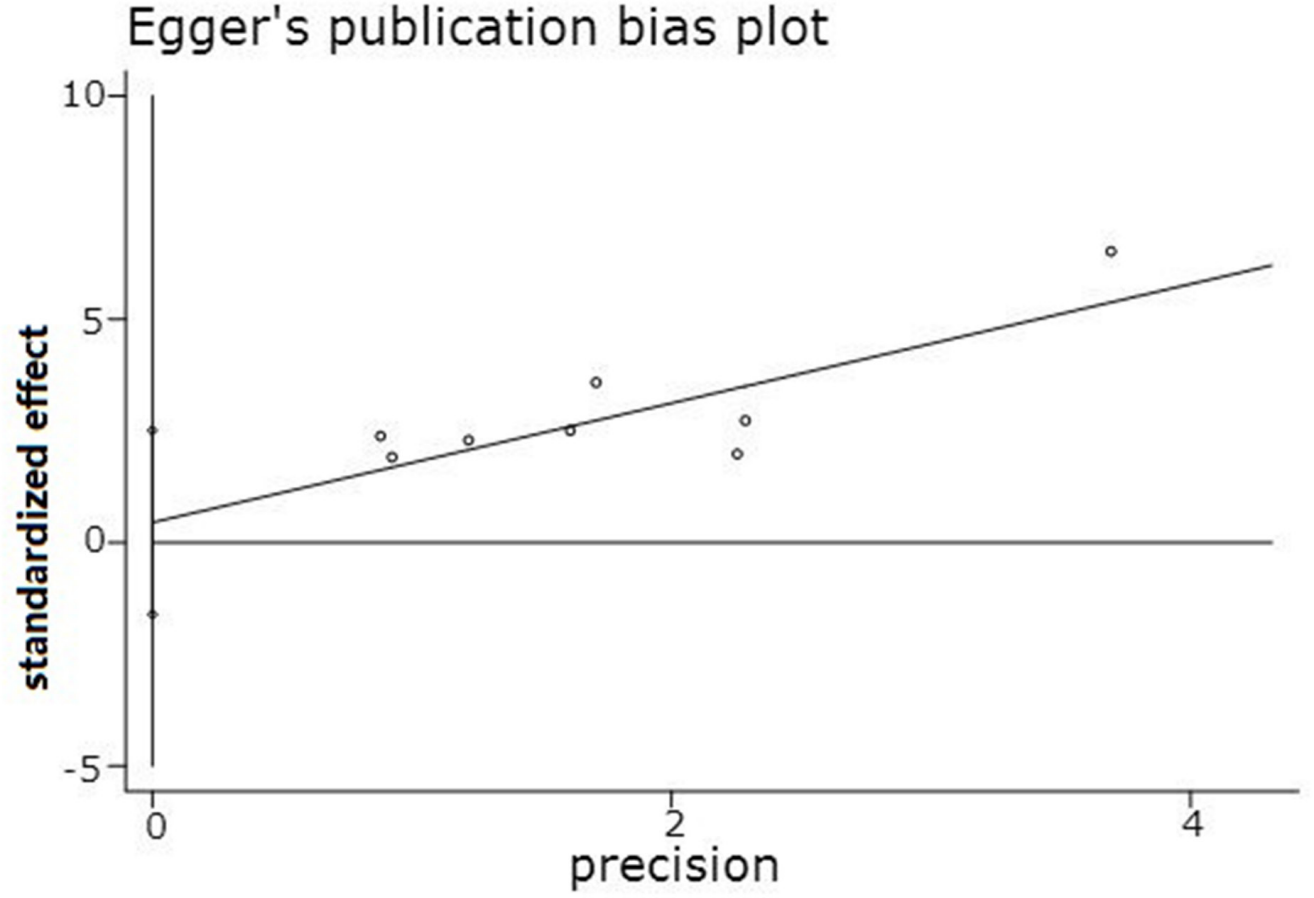
Egger’s publication bias plot

For the meta-analysis of the association between the miR-10b expression level and LNM occurrence, the funnel plot was slightly asymmetrical, as shown in Figure 4. Additionally, the statistical assessment of publication bias using the Begg’s test (*Pr*>|*z*|=0.266) and the Egger’s test (*P*>|*t*|=0.609, 95% CI: -1.602 to 2.509), as shown in Figure 5, revealed no publication bias (*P*>0.05). The trim and fill method was also adopted to test for publication bias, and the results of this test suggested that the meta-analysis results were identical.

### Sensitivity analysis

For the meta-analysis of the association between *the* miR-10b expression level and LNM occurrence, a sensitivity analysis was performed by deleting each study in turn from the pooled analysis. It aimed to test the influence of the removed data set on the overall ORs. The result was not significantly influenced by the exclusion of any of the studies, suggesting that the result of the synthetic analysis was robust.

## DISCUSSION

The LNM is the most common metastasis pathway in most cancers, and distant metastasis often occurs in the advanced stages of cancer. LNM and DM are positively important for diagnosis in tumor–node–metastasis staging and the treatment strategy for cancer patients, as well as indicators for predicting prognosis. Accordingly, there is a need to identify new, prognostic biomarkers for cancer to establish prognostic sets needing different therapeutic intensity.

MiRNAs are members of a family of endogenous, highly conserved, small non-coding RNA molecules (18–24 nucleotides) that regulate the expression of a wide variety of genes and have similar physiological functions [19]. Through base pairing with the 3’-untranslated region (3’-UTR) of target genes, miRNAs enhance mRNA degradation or inhibit posttranscriptional translation [20]. miRNAs can direct a wide variety of normal biological mechanisms, such as embryonic development, cell growth, apoptosis and differentiation. MiRNAs have been found to regulate both oncogenes, such as ras, and tumor suppressor genes, such as PTEN [21, 22]. Although miRNAs have been widely investigated in recent years, the molecular regulatory mechanisms of miRNAs and their roles in the development of cancers remain largely unknown and need further investigation.

MiR-10b has been shown to be dysregulated in certain types of cancer and to play an important role in invasion and metastasis [7–9, 23, 24]. However, the exact mechanism of miR-10b in cancer is not completely understood. Overexpression of miR-10b was shown to exert multiple effects on various cancers, through different mechanisms. In addition, miR-10b was shown to be highly expressed in metastatic breast cancer cell lines as well as in patients’ metastatic breast tumors. Upregulation of miR-10b, which is induced by Twist, inhibits the translation of HOXD10, a transcription factor known for its roles in cell motility, resulting in increased expression of the prometastatic gene RHOC [24]. Silencing of miR-10b, both *in vitro* and *in vivo*, with antagomirs significantly decreases miR-10b levels and increases the levels of HOXD10, thereby inhibiting metastasis [25].

MiR-10b also plays an important role in the invasion and metastasis of nasopharyngeal carcinoma cells. For instance, after transfection of the miR-10b inhibitor, the ability of nasopharyngeal carcinoma cell lines to migrate and invade was reduced [26]. Also, through regulation of Kruppel-like factor 4 (KLF4) expression in human esophageal cancer cell lines, miR-10b was found to induce tumor migration and invasion [27]. Additionally, the loss of CADM1, a tumor suppressor gene involved in cell–cell interactions and epithelial-like phenotype, can contribute to cancer invasion and metastasis in epithelial-derived cancers by inducing epithelial mesenchymal transition (EMT) [4, 28, 29]. Indeed, downregulation or loss of CADM1 expression is frequently observed in various cancers, such as NSCLC, HCC, cervix, prostate and pancreas cancer [30–35], especially in those with LNM and DM [36]. Multiple studies showed that miR-10b regulates CADM1 expression at the post transcriptional level. Loss of Cadherin 1 (CDH1), a glycoprotein responsible for calcium-dependent cell-to-cell adhesion [37], can also induced EMT, and its downregulation is associated with tumor grade and stage and contributes to the transition of adenoma to carcinoma in animal models [38]. The research by Zhang et al. revealed that the CDH1mRNA and protein were overexpressed in miR-10b-suppressed NSCLC cells compared with controls, suggesting that miR-10b might be necessary to drive the expression of CDH1 [39]. In addition, target genes, such as MMP-9, vimentin (VIM), and CDH1, which were found to be differently expressed in the nasopharyngeal carcinoma cells transfected with miR-10b mimics and with miR-10b inhibitors, were also involved in migration and invasion. Moreover, miR-10b was also confirmed to be overexpressed in malignant glioma, hepatocellular carcinoma, and pancreatic cancer [40, 41].

This study demonstrated that the high expression of miR-10b was significantly correlated with DM and regional lymph node involvement. This indicated that overexpression of miR-10b was closely related to the aggressive behavior of tumor cells, thus suggesting miR-10b as a promising biomarker to predict the prognosis of cancer patients. In future experiments, the exact mechanism whereby miR-10b exerts its effects on promoting tumor cell invasion and metastasis will be further investigated.

In assessing the relationship between miR-10b expression and LNM, the heterogeneity tests revealed mild heterogeneity. The heterogeneity may originate from different tumor types and different definitions of high expression of miR-10b. At the same time, some small sample studies may also be the cause of heterogeneity. Additionally, the proportion of advanced tumors in different research centers may vary, and can also be a source of heterogeneity.

Since most of the studies enrolled lacked miR-10b expression data based on different ages and tumor stages, no meaningful subgroup analysis was conducted.

Almost all relevant studies were browsed in full text and carefully evaluated. Unfortunately, in many studies, patients (especially those of the European and African populations) were not grouped according to the level of miR-10b expression to described their clinicopathological parameters. Many researchers detected tissue levels of miR-10b, based on LNM and DM. Thus, there is a lack of pathological parameters related to metastasis based on different levels of miR-10b expression, and more data are needed to verify the results of this study in the future.

## MATERIALS AND METHODS

### Literature retrieval strategy

To obtain the potentially eligible studies, systematic online literature search was conducted against multiple databases, including PubMed, Springer, Google Scholar, Gene expression Omnibus, China National Knowledge Infrastructure (CNKI), Chongqing VIP Information Network, and Wanfang. The keywords for the search were as follows: “miR-10b”, “microRNA-10b”, “Prognosis”, “Clinicopathology” and “metastasis”. The relevant documents found were retrieved, using April 1, 2017, as the retrieval period deadline. In addition, other relevant articles were also obtained by manually viewing the reference list.

### Inclusion and exclusion criteria

The inclusion criteria for the articles were as follows: 1) the role of miR-10b in the development of human carcinoma was investigated; 2) the expression level of miR-10b in primary cancerous tissue was measured; 3) patients were grouped according to the expression level of miR-10b; 4) related clinicopathologic parameters were reported in the study. The exclusion criteria for the articles were as follows: 1) duplicate study publications; 2) studies without valuable data or data acquired through animal experiments; 3) reviews, letters, case reports, and expert opinions; 4) the expression level of miR-10b is detected in serum.

### Date extraction and quality assessment

The data and information from all eligible studies were independently extracted by two investigators (RB Liao and BX Tong) through cross-check. The following data and information were collected from each study: author, publication year, country, race, cancer type, total number of patients, number of patients with high miR-10b expression in the group and with low miR-10b expression in the group, number of patients with LNM and DM in each group, and the criteria for high miR-10b expression. If there were disagreements, a consensus was reached by a third investigator (HJ Wu). The Newcastle-Ottawa Scale (NOS) was applied to assess the quality of all included studies. The NOS scores ranged from 0 to 9, and the study with an NOS score ≥ 6 was considered to be of high quality. The quality of all studies included in this meta-analysis was varied from 5 to 9, with a mean value of 6.7.

### Statistical methods

The present meta-analysis was conducted using the RevMan5.2 software and Stata SE12.0 software. The heterogeneity among the enrolled studies was determined by the chi-square-based Q-test and I^2^ statistics; a P-value for the Q-test <0.05 and a I^2^-value>50% were considered as indicators of severe heterogeneity. The random-effects model was applied to the studies with a significant heterogeneity (P_Q_≤0.05, I^2^≥50%). Otherwise, the fixed-effects model was adopted (P_Q_>0.05, I^2^<50%). Potential publication bias was assessed using a funnel plot, and the sensitivity analysis was also performed to ensure the reliability of the results. The P-value <0.05 was considered statistically significant.

## CONCLUSION

This meta-analysis has analyzed the association between the miR-10b expression levels with LNM and DM occurrence in patients with carcinoma. Compared with their incidence in those with low miR-10b expression, the incidence of LNM and DM was higher in cancer patients with high miR-10b expression. However, there were also some limitations in this meta-analysis. First, the number of included cancer patients was still relatively small, thus larger and better designed studies would be necessary to confirm the results obtained in this meta-analysis. Also, all patients included in this meta-analysis were Asian, and no patients of other races could be included. Additionally, potential publication bias may exist, which may originate from unpublished negative results or the small sample studies included, although no significant publication bias was observed based on the trim and fill method and the sensitivity analysis also showed the results were robust. Furthermore, the criterion for high miR-10b expression was varied in these studies. Therefore, larger size, multicenter, and higher quality studies are needed for further research, based on a unique criterion to classify the miR-10b expression groups.
